# Rooperol, an inhibitor of cytokine synthesis, decreases the respiratory burst in human and rat leukocytes and macrophages

**DOI:** 10.1080/09629359791938

**Published:** 1997-02

**Authors:** A. Guzdek, B. Turyna, A. C. Allison, K. Sladek, A. Koj

**Affiliations:** 1 Institute of Molecular Biology Jagiellonian University Cracow 31-120 Poland; 2 DAWA Inc. Belmont CA 94002 USA; 3 Institute of Internal Medicine Collegium Medicum Jagiellonian University Cracow 31-066 Poland

## Abstract

Luminol-enhanced chemiluminescence was measured in fresh whole human blood, or human neutrophils isolated from heparinized blood, human alveolar macrophages and rat alveolar macrophages stimulated with bacterial endotoxin (LPS). Tetraacetate esters of rooperol, a dicatechol showing anticytokine activity, added to cells simultaneously with LPS inhibited the respiratory burst. The effective concentrations of rooperol were in the range of 1-10 μM depending on cell type and corresponded well with inhibition of nitric oxide production by rat alveolar macrophages. Thus rooperol may reduce some effects of excessive phagocytic activity and inflammatory reaction but by quenching free radicals production may also diminish the resistance to bacterial infections.


Mediators of Inflammation, 6, 53–57 (1997)

### Rooperol, an inhibitor of cytokine synthesis, decreases the respiratory burst in human and rat leukocytes and macrophages

A. Guzdek,[1] B. Turyna,[1] A. C. Allison,[2] K. Sładek[3] and A. Koj[1,CA]

[1]Institute of Molecular Biology, Jagiellonian University, 31-120 Cracow, Poland;[2] DAWA Inc., Belmont, CA 94002, USA; [3]Institute of Internal Medicine, Collegium Medicum, Jagiellonian University, 31-066 Cracow, Poland

[CA]Corresponding Author
Fax: (+48) 12 336907

**LUMINOL-ENHANCED chemiluminescence was measured in fresh whole human blood, or human neutrophils isolated from heparinized blood, human alveolar macrophages and rat alveolar macrophages stimulated with bacterial endotoxin (LPS). Tetraacetate esters of rooperol, a dicatechol showing anticytokine activity, added to cells simultaneously with LPS inhibited the respiratory burst. The effective concentrations of rooperol were in the range of 1–10 μM depending on cell type and corresponded well with inhibition of nitric oxide production by rat alveolar macrophages. Thus rooperol may reduce some effects of excessive phagocytic activity and inflammatory reaction but by quenching free radicals production may also diminish the resistance to bacterial infections.**

**Key words:** Chemiluminescence, Endotoxin, Nitric oxide production, Phagocytes, Respiratory burst

### Introduction

Phagocytes from blood, i.e. polymorphonuclear leukocytes and monocytes, and tissue macrophages, are involved in complex defence mechanisms against pathogenic bacteria. During phagocytosis they exhibit increased metabolic activity known as respiratory burst which generates superoxide as its primary product. Superoxide is formed in effect of oxygen reduction by NADPH-oxidase, a multicomponent enzyme present in the cell membrane of phagocytes.[1] In a series of further reactions, superoxide gives rise to reactive oxygen intermediates (ROI) such as hydrogen peroxide, hydroxyl radical and hypochlorous acid which serve as microbicidal agents but produced in excess may damage host cells.[2] Respiratory burst is induced not only by intact bacteria but also by opsonized particles, endotoxin, some cytokines (tumour necrosis factor, TNFα, interferon-γ, IFN-γ), N-formylated chemoattractant peptides and activators of kinase C (phorbol esters).[1] Bactericidal activity is also generated by reactive nitrogen intermediates, such as nitric oxide (NO)[3] possessing a broad spectrum of biological activities and produced in macrophages by inducible nitric oxide synthase (iNOS).[4] The interaction between reactive oxygen and nitrogen intermediates leads to peroxynitrite and may increase cytotoxicity or inflammation.[3] Thus it is not surprising that simultaneous inhibition of nitric oxide synthase and NADPH oxidase suppress inflammatory arthritis.[5] Moreover, some antioxidants show anti-inflammatory properties and inhibit synthesis of acute phase cytokines.[6,7] We recently demonstrated that rooperol, a plant dicatechol, decreases production of tumour necrosis factor (TNFα), interleukin-1 (IL-1) and interleukin-6 (IL-6) in LPS-stimulated human and rat macrophages and NO production in rat macrophages.[7,8] Here we describe the effect of rooperol tetraacetate on the respiratory burst in human and rat phagocytes.

### Materials and Methods

### Reagents

Mono-Poly resolving medium was obtained from ICN Biomedicals, Meckenheim, Germany. RPMI and antibiotics-antimycotics came from Gibco Life Technologies Inc., Grand Island, NY, USA. Fetal bovine serum (FBS), lipopolysaccharide from *Escherichia coli* 026:B6 (LPS), luminol (5-amino-2,3-dihydro-1,4-phthalazinedione), latex beads (1 μm), glucose were purchased from Sigma, St Louis, MO, USA. Rooperol tetraacetate (RTA) was kindly provided by Dr P. B. Kruger, ESP Group, Department of Pharmacology, Uni-

© 1997 Rapid Science Publishers versity of Stellenbosch, South Africa. All other reagents obtained from POCH, Gliwice, Poland were of highest purity grade.

### Cells, isolation and culture

Twenty μl of whole bood was taken from the finger of healthy volunteers (25–48 years of age) and used immediately for respiratory burst measurement. Neutrophils were isolated from fresh heparinized human blood of healthy donors by centrifugation with Mono-Poly resolving medium, washed in Hanks' Balanced Salt Solution (HBSS) without $Ca^{2+}$ and c. 5000 cells were used for experiments. Human lung macrophages were obtained by fibre-optic bronchoscopy of patients examined for diagnostic purposes following the guidelines of the American Thoracic Society.[9] The cells were collected in sterile phosphate buffered saline (PBS), centrifuged, washed in PBS and c. 5000 cells were used for each experiment. The lungs of killed by ether overdose rats were lavaged with 3 × 10 ml of cold PBS. After centrifugation, the cell pellet was washed in cold PBS and $2 \times 10^4$ cells were used for respiratory burst estimation.

NO production by rat alveolar macrophages was evaluated in 24 h culture of the cells. Isolated rat macrophages were seeded on 12-well plates (Costar) in RPMI with 8% fetal bovine serum and antibiotics. After 2 h the unattached cells were discarded, and the adherent cells were cultured for 24 h. Then rooperol tetraacetate (RTA) and/or LPS (500 ng/ml) in RPMI containing 2% FBS was added. NO was estimated in the medium collected after 24 h culturing the cells with the drugs.

### Estimation of chemiluminescence (CL) and NO production

Whole blood in HBSS containing glucose and 0.5% FCS was incubated for 10 min with luminol (1.4 mM), RTA (0.6, 2, 5 and 10 μM) and/or 10 ng/ml LPS. The addition of 50 μl of latex beads ($2 \times 10^{10}$) was the start point of chemiluminescence estimation in Scintillation Spectrometer Rackbeta 1211, LKB.[10] Neutrophils and macrophages were incubated with luminol, RTA and/or LPS for 20 min. As a result of exposure to latex beads respiratory burst occurs and its intensity depends on phagocytes priming by LPS.

NO production was estimated with Griess reagent and nitrite concentration was determined by using dilutions of sodium nitrite in water as a standard.[11] Rooperol tetraacetate at 20 μM concentration had no effect on nitrite assay.

### Statistical analysis

Data were analysed by paired Student's *t*-test.

### Results

### Effect of rooperol on the respiratory burst of human cells

Addition of bacterial endotoxin (10 ng/ml) to fresh human blood or isolated neutrophils followed by latex beads increased chemiluminescence within the first 15–20 min (Table 1) and a decline occurred usually around 30–40 min after the addition of latex beads. However, considerable individual variations were observed, especially with full blood, in which the maximum was often delayed or prolonged (Fig. 1). When rooperol tetracetate was added to fresh human blood simultaneously with LPS a marked reduction of respiratory burst was found (Fig. 1a and 1ax) and occasionally the chemiluminescence was reduced below control (Fig. 1ax). Similar inhibitory effects of rooperol were also visible with neutrophils isolated from human peripheral blood (Fig. 1b) and human alveolar macrophages (Fig. 1c). These effects were dose-dependent and for human neutrophils and macrophages almost total inhibition of respiratory burst occurred at 10 μM RTA (Fig. 1b and 1c). In the case of full blood or isolated neutrophils 10 μM RTA reduced latex-beads-induced chemiluminescence by approximately three-fold (Fig. 2). A similar inhibition of chemiluminescence was observed in one experiment with isolated human blood monocytes (data not shown).

**Table 1**. Stimulatory effect of LPS (10 ng/ml) on latex-beads-induced chemiluminescence of cells present in 20 μl of fresh human blood or isolated neutrophils (~5000 cells). The chemiluminescence was recorded after 3, 9, 15 and 21 min following the addition of latex beads. The results are reported as percentage of control preparation (without LPS) assumed as 100% at any given time in each experiment: mean ±SD of between three and eight independent experiments. Statistically significant differences at $^{*}P < 0.05$, $^{**}P < 0.01$, $^{+}P < 0.005$ and $^{++}P < 0.002$. NS, not significant

| Cells | Time (min) | | | |
|---|---|---|---|---|
| | 3 | 9 | 15 | 21 |
| Full blood | 132 ± 43** | 163 ± 45+ | 191 ± 41+ | 242 ± 92* |
| Neutrophils | 128 ± 28(NS) | 176.29* | 191.16+ | 161 ± 27++ |

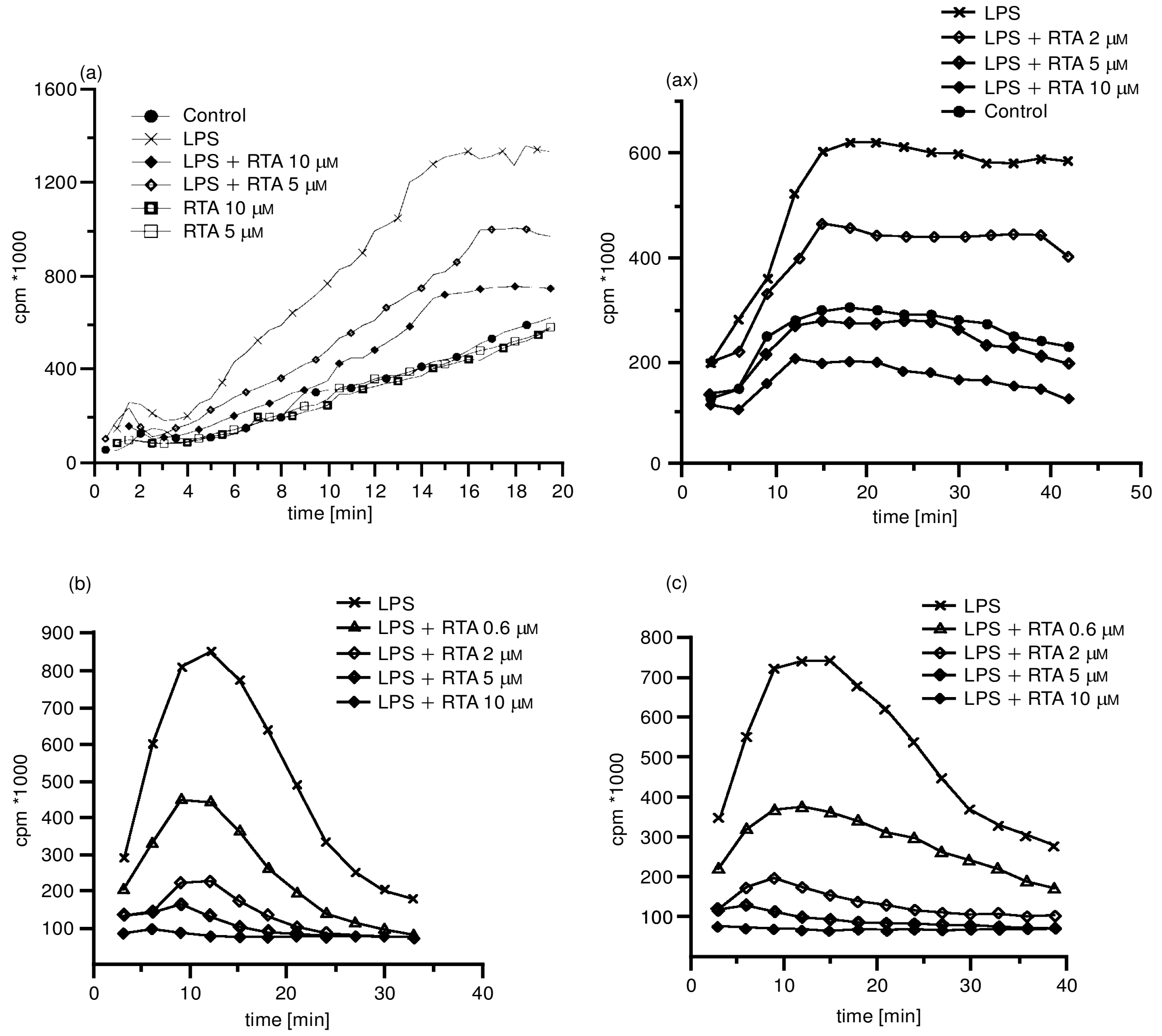

FIG. 1. The inhibitory effect of RTA (0.6, 2, 5 and 10 μM) on chemiluminescence induced by LPS (10 ng/ml) and latex beads suspension (50 μl) in white cells present in 20 μl of full human blood (a and ax); in 5000 neutrophils isolated from peripheral human blood (b); or in 5000 human alveolar macrophages (c). Fresh full blood was preincubated with luminol, RTA and/or LPS for 10 min, isolated neutrophils and macrophages for 20 min, and then latex beads were added and chemiluminescence measured.

### Effect of rooperol on the respiratory burst and NO production by rat macrophages

The respiratory burst of rat alveolar macrophages primed with LPS was also quenched by rooperol (Fig. 3). In the conditions used, rat alveolar macrophages response to RTA was less pronounced in comparison with human macrophages. Moreover, the maximal response to 10 ng/ml of LPS occurred faster (in about 5 min after phagocytosis initiation) but also declined earlier (Fig. 3). When rat alveolar macrophages were cultured for 24 h with 500 ng/ml of LPS they produced significant amounts of nitric oxide determined as nitrite (123.8 ± 17.8 nmol/ml, $n = 6$). Rooperol reduced NO production in a dose-dependent manner (Fig. 4), the $IC_{50}$ was found to be between 5 and 10 μM.

### Discussion

Acetate esters of rooperol, a dicatechol from the South African plant *Hypoxis rooperi*, were shown to inhibit production of TNFα, IL-1β and IL-6 in endotoxin-stimulated human alveolar macrophages, blood monocytes and U937 promonocytic cell line.[7] Rooperol tetraacetate (RTA) was effective in low micromolar range ($IC_{50}$ = 10–20 μM) and at this concentration had no effect on cell viability as shown by the incorporation of $^{14}C$-leucine into macro-

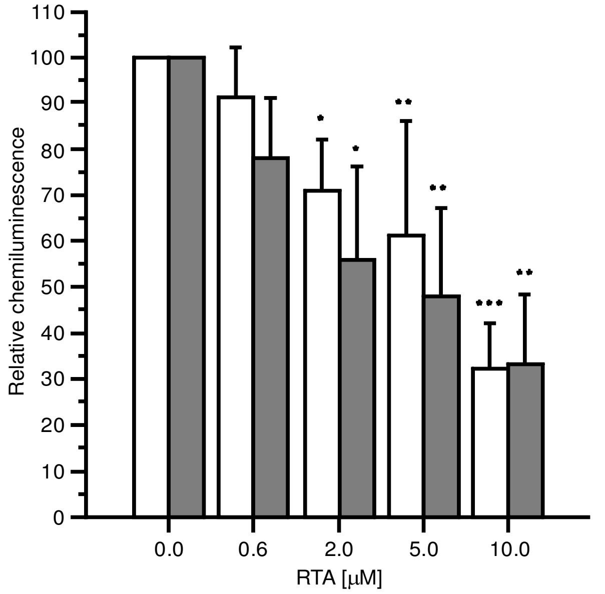

FIG. 2. Dose-dependent inhibition of LPS-stimulated chemiluminescence of cells from 20 μl of whole blood (empty bar) and neutrophils (5000 cells) (striped bar), 9 min after latex addition. Full blood was preincubated for 10 min, neutrophils for 20 min with luminol, RTA and/or LPS. Chemiluminescence caused by LPS alone was assumed as 100%. Mean (±SD) of three to six experiments. Statistically significant differences at $^{*}P < 0.05$, $^{**}P < 0.02$ and $^{***}P < 0.01$.

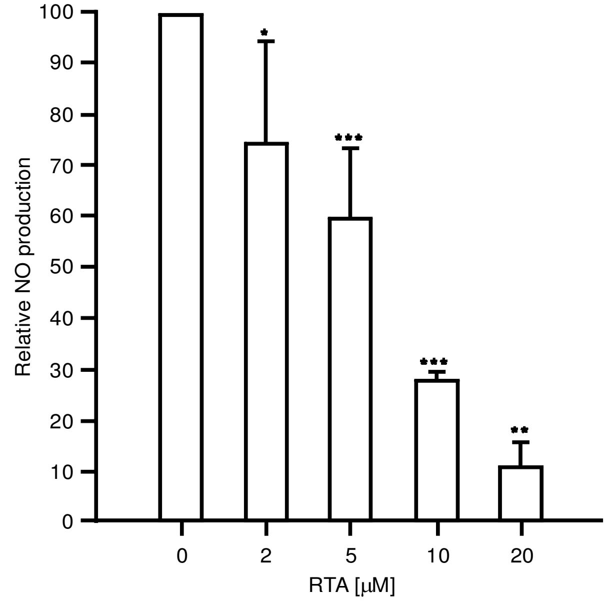

FIG. 4. Percentage inhibition of LPS-stimulated (500 ng/ml) NO production by rat alveolar macrophages cultured for 24 h with increasing concentrations of RTA. Mean (±SD) of five to seven experiments. Statistically significant differences at $^{*}P < 0.05$, $^{**}P < 0.005$ and $^{***}P < 0.001$.

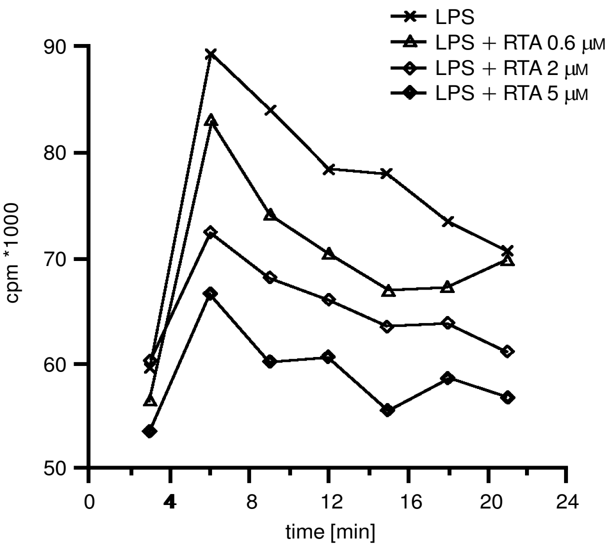

FIG. 3. Effect of RTA (1, 2.5 and 5 μM) on chemiluminescence of $2 \times 10^4$ rat alveolar macrophages stimulated with 10 ng/ml LPS. For further explanations see legend to Fig. 1.

phage proteins and cellular contents of lactate dehydrogenase. Subsequent studies revealed that RTA may inhibit cytokine synthesis in promonocytic U937 cell line at the pretranslational level.[8] All those results suggested that rooperol esters are potential anti-inflammatory drugs.

The experiments described here clearly demonstrate another anti-inflammatory activity of rooperol, i.e. inhibition of the respiratory burst. We found that RTA at micromolar concentrations significantly decreased chemiluminescence of human blood leukocytes and alveolar macrophages, as well as of rat alveolar macrophages, that had been primed with LPS and stimulated to phagocytosis by latex beads. The mechanism of respiratory burst inhibition by rooperol is not elucidated but may involve events related to LPS priming,[12] direct interaction with enzymatic pathway of oxygen radicals formed by NADP oxidase,[1] or involvement of cell membrane constitutents including protein kinase C as described for coenzyme Q[13] which is structurally related to rooperol.

Our results confirm earlier observations that RTA can also inhibit NO production in rat alveolar macrophages[7] and murine endothelial cells.[14] The broad spectrum of biological activities of rooperol suggests that this compound may be beneficial in the treatment of various inflammatory diseases, such as asthma or rheumatoid arthritis, in which overproduction of acute phase cytokines, nitric oxide and reactive oxygen intermediates has been reported.[5,15] It should be remembered, however, that by quenching free radicals production rooperol may also diminish the resistance of organism to bacterial infections.[16]

### References

1. Maly FE, Schurer-Maly CC. How and why cells make superoxide: the 'phagocytic' NADPH oxidase. *News Physiol Sci* 1995; **10**: 233–238.
2. Schreck R, Albermann K, Bauerle PA. Nuclear factor κB: an oxidative stress-responsive transcription factor of eukaryotic cells. *Free Rad Res Commun* 1992; **17**: 221–237.
3. Kaplan SS, Lancaster JR Jr, Basford RE, Simmons RL. Effect of nitric oxide on staphyloccocal killing and interactive effect with superoxide. *Infect Immun* 1996; **64**: 69–76.
4. Kroncke KD, Fehsel K, Kolb-Bachofen V. Inducible nitric oxide synthase and its product nitric oxide, a small molecule with complex biological activities. *Biol Chem Hoppe-Seyler* 1995; **376**: 327–343.
5. Miesel R, Kurpisz M, Kroeger H. Suppression of inflammatory arthritis by simultaneous inhibition of nitric oxide synthase and NADPH oxidase. *Free Radical Biol Med* 1996; **20**: 75–81.
6. Eugui EM, DeLustro B, Rohafza S, Ilnicka M, Lee SW, Wilhelm R, Allison AC. Some antioxidants inhibit, in a co-ordinate fashion, the production of tumor necrosis factor-α, IL-1β, and IL-6 by human peripheral blood mononuclear cells. *Int Immun* 1994; **6**: 409–422.
7. Guzdek A, Niżankowska E, Allison AC, Kruger PB, Koj A. Cytokine production in human and rat macrophages and dicatechol rooperol and esters. *Biochem Pharmacol* 1996; **52**: 991–998.
8. Guzdek A, Rokita H, Cichy J, Allison AC, Koj A. Rooperol tetraacetate, inhibitor of cytokine synthesis, decreases TNFα, IL-1β and IL-6 mRNA levels and binding activity of transcription factors in U937 cells. *Biochem Pharmacol* (submitted).
9. American Thoracic Society Guidelines. Summary and recommendation of a workshop on the investigational use of fiberoptic bronchoscopy and bronchoalveolar lavage in asthmatics. *Am Rev Resp Dis* 1985; **132**: 180–182.
10. Turyna B, Milc J, Łaczka A, Cholewa K, Łaczka M. Biocompatibility of glass-crystalline materials obtained by the sol-gel method: effect on macrophage function. *Biomaterials* 1996; **17**: 1379–1386.
11. Ding AH, Nathan CF, Stuehr DJ. Release of reactive nitrogen intermediates and reactive oxygen intermediates from mouse peritoneal macrophages: comparison of activating cytokines and evidence for independent production. *J Immunol* 1988; **141**: 2407–2410.
12. Landmann R, Scherer F, Schumann R, Link S, Sansano S, Zimmerli W. LPS directly induces oxygen radical production in human monocytes via LPS binding protein and CD14. *J Leukocyte Biol* 1995; **57**: 440–449.
13. Kanno T, Utsumi T, Takehara Y, Ide A, Akiyama J, Yoshioka T, Horton AA, Ustumi K. Inhibition of neutrophil superoxide generation by alpha-tocopherol and coenzyme Q. *Free Radical Res* 1996; **24**: 281–289.
14. Bereta J, Bereta M, Allison AC, Kruger PB, Koj A. Inhibitory effect of di-catechol rooperol on VCAM-1 and iNOS expression in cytokine-stimulated epithelium. *Life Sci* 1997; **60**: 325–334.
15. Barnes PJ, Liew FY. Nitric oxide and asthmatic inflammation. *Immunol Today* 1995; **16**: 128–130.
16. Evans TJ, Buttery LDK, Carpenter A, Springall DR, Polak JM, Cohen J. Cytokine-treated human neutrophils contain inducible nitric oxide synthase that produces nitration of ingested bacteria. *Proc Natl Acad Sci USA* 1996; **93**: 9553–9558.

ACKNOWLEDGEMENTS. This work was supported in part by grant no. 1227/P05/95/08 to A.K. from the Committtee of Scientific Research (KBN, Warsaw, Poland).

**Received 24 October 1996;**
**accepted in revised form 27 November 1996**

## References

[PDF_00472] Barnes P. J., Liew F. Y. (1995). Nitric oxide and asthmatic inflammation.. Immunol Today.

[PDF_00469] Bereta J., Bereta M., Allison A. C., Kruger P. B., Koj A. (1997). Inhibitory effect of di-catechol rooperol on VCAM-1 and iNOS expression in cytokine-stimulated endothelium.. Life Sci.

[PDF_00458] Ding A. H., Nathan C. F., Stuehr D. J. (1988). Release of reactive nitrogen intermediates and reactive oxygen intermediates from mouse peritoneal macrophages. Comparison of activating cytokines and evidence for independent production.. J Immunol.

[PDF_00440] Eugui E. M., DeLustro B., Rouhafza S., Ilnicka M., Lee S. W., Wilhelm R., Allison A. C. (1994). Some antioxidants inhibit, in a co-ordinate fashion, the production of tumor necrosis factor-alpha, IL-beta, and IL-6 by human peripheral blood mononuclear cells.. Int Immunol.

[PDF_00474] Evans T. J., Buttery L. D., Carpenter A., Springall D. R., Polak J. M., Cohen J. (1996). Cytokine-treated human neutrophils contain inducible nitric oxide synthase that produces nitration of ingested bacteria.. Proc Natl Acad Sci U S A.

[PDF_00444] Guzdek A., Nizankowska E., Allison A. C., Kruger P. B., Koj A. (1996). Cytokine production in human and rat macrophages and dicatechol rooperol and esters.. Biochem Pharmacol.

[PDF_00466] Kanno T., Utsumi T., Takehara Y., Ide A., Akiyama J., Yoshioka T., Horton A. A., Utsumi K. (1996). Inhibition of neutrophil-superoxide generation by alpha-tocopherol and coenzyme Q.. Free Radic Res.

[PDF_00431] Kaplan S. S., Lancaster J. R., Basford R. E., Simmons R. L. (1996). Effect of nitric oxide on staphylococcal killing and interactive effect with superoxide.. Infect Immun.

[PDF_00434] Kröncke K. D., Fehsel K., Kolb-Bachofen V. (1995). Inducible nitric oxide synthase and its product nitric oxide, a small molecule with complex biological activities.. Biol Chem Hoppe Seyler.

[PDF_00462] Landmann R., Scherer F., Schumann R., Link S., Sansano S., Zimmerli W. (1995). LPS directly induces oxygen radical production in human monocytes via LPS binding protein and CD14.. J Leukoc Biol.

[PDF_00437] Miesel R., Kurpisz M., Kroger H. (1996). Suppression of inflammatory arthritis by simultaneous inhibition of nitric oxide synthase and NADPH oxidase.. Free Radic Biol Med.

[PDF_00428] Schreck R., Albermann K., Baeuerle P. A. (1992). Nuclear factor kappa B: an oxidative stress-responsive transcription factor of eukaryotic cells (a review).. Free Radic Res Commun.

[PDF_00455] Turyna B., Milc J., Laczka A., Cholewa K., Laczka M. (1996). Biocompatibility of glass-crystalline materials obtained by the sol-gel method: effect on macrophage function.. Biomaterials.

